# A Stakeholder Perspective on Ethical Leadership in Sport: Bridging the Gap Between the Normative and Descriptive Lines of Inquiry

**DOI:** 10.5334/pb.543

**Published:** 2020-12-14

**Authors:** Bram Constandt, Leonie Heres, Mathieu Marlier, Annick Willem

**Affiliations:** 1 Department of Movement and Sports Sciences, Ghent University, BE; 2 Utrecht University School of Governance, Utrecht University, NL; 3 Department of International Sport Management, LUNEX University, LU

**Keywords:** descriptive leadership, ethical leadership, normative leadership, sport ethics, sport integrity, stakeholder perspective

## Abstract

This critical PhD review paper examines existing scholarship on ethical leadership in sport. Following a general trend in business ethics and related fields, ethical leadership has gained considerable research attention in sport over the last decades. Within this growing body of literature, ethical leadership is often presented as part of the desired strategic response of sport organizations to tackle the so-called dark side of sport (i.e., formed by such ethical issues as abuse, violence, management fraud, match-fixing, and doping). However, this critical PhD review paper argues that the current body of literature on ethical leadership in sport has matured along two strongly related yet quite isolated lines of inquiry: a normative (i.e., philosophical) and a descriptive (i.e., empirical) line. While the normative line of inquiry focuses on what ethical leadership in sport should look like based on moral reasoning, the descriptive line examines how ethical leadership in sport is perceived in practice and how it relates to certain antecedents and outcomes. As both lines offer complementary insights, we advocate future research to bridge this gap to come to an improved understanding of ethical leadership in sport. To this aim, we propose a broad stakeholder perspective on ethical leadership in sport, in which necessary attention is given to how all involved stakeholders make sense of ethical leadership as a socially constructed and context-dependent phenomenon.

## Introduction

Leadership continues to be one of the most studied social phenomena. Although many definitions exist, leadership can be understood as a process or relationship between a leader and follower(s) (in a narrow, hierarchical view) or stakeholder(s) (in a broad, less hierarchical view), aiming to accomplish shared goals (Yukl, 2008). Within the extensive body of literature on leadership, there is consensus that leadership entails an inherent ethical dimension (Ciulla et al., 2018). Ethics^1^ are at “the heart of leadership” (Ciulla et al., 2018). In fact, formal forms of leadership imply some extent of authority, which causes leaders’ decisions to have a broad impact and increases the likelihood that ethical issues or dilemmas occur (Flanigan, 2018; Heres, 2014).

Sport is a social context in which the ethical dimension of leadership is amply illustrated. Leaders in sport, such as coaches, managers, and board members have a strong impact on the people they are working with (e.g., players). Moreover, certain characteristics of competitive sport, such as a focus on performing and excellence, and the presence of often high levels of emotional involvement and pressure, add to an environment in which many different types of ethical challenges occur (Bortoli et al., 2012; Vanden Auweele et al., 2016). These ethical challenges – including but not limited to violence, (sexual) abuse, (management) fraud, doping, and match-fixing – have led to a certain tension between sport’s dark side on the one hand – formed by these challenges – and its enormous social potential as stimulator of physical and mental health and social cohesion on the other hand (Cashmore & Cleland, 2014; Ordway & Opie, 2017). Against this background, ethical leadership in sport has been put forward as part of the desired strategic response of sport organizations to mitigate this tension, ensuring that the positive aspects of sport prevail (Constandt, 2019). In other words, ethical leadership in sport might help to stimulate and protect the integrity of sport (and those involved), while operating as a positive force when it comes to socializing certain values (e.g., trust and respect) and norms in society (Claringbould et al., 2018).

As a consequence, research attention for ethical leadership in sport has increased over the past years (Welty Peachey et al., 2015). Most studies have applied a normative or a descriptive view on ethical leadership. Philosophical, normative studies focus on what ethical leadership in sport *is* and *should look like* (i.e., prescriptions of ethical leadership), based on moral reasoning. Descriptive studies emphasize how ethical leadership *is perceived* in practice (i.e., perceptions of ethical leadership), based on empirical study designs. Whereas both views on ethical leadership are considered “complementary sides of the same coin” (see Flanigan, 2018), they have evolved in quite isolated terms. This is unfortunate, given that the integration of both views is needed to come to a comprehensive and more holistic consideration of ethical leadership. Furthermore, both views are also constantly influencing each other in practice and everyday life, as perceptions of ethical leadership impact prescriptions, and the other way around (Flanigan, 2018).

Despite its relevance, the understandings of studies on ethical leadership in sport have not yet fully trickled down to the actual world of sport, as illustrated in many sport governing bodies and clubs. More precisely, ongoing reports of ethical scandals (e.g., abuse in gymnastics, match-fixing in football and tennis, state-sponsored doping schemes in relation to the Olympics) in the international sporting world indicate a lack of ethical leadership, as well as a paucity of concrete efforts to enable and stimulate ethical leadership in sport (Burton et al., 2017; Tomlinson, 2014). A potential explanation for this gap between research and practice might relate to the limited practical applicability of existing ethical leadership studies, which often lack context-specific recommendations to actually deal with values of (and in) sport and the dilemmas that occur (Constandt, 2019; Grange, 2014). As such, a more comprehensive approach of ethical leadership in sport is encouraged, in which normative and descriptive insights are integrated to shed more light on such questions as what ethical leadership is, how it is developed, whom it concerns, and how it can be stimulated. In other words, such integration of both types of insights would help to further expose “the true depth” of ethical leadership (Price, 2018, p. 687).

Attempting to integrate the normative and descriptive lines of ethical leadership research by means of a more comprehensive approach is not completely novel. However, existing studies in this area have led to little follow-up research and limited utilization in practice (Eisenbeiß, 2012; Frisch & Huppenbauer, 2014; Giessner & Van Quaquebeke, 2010; Heres, 2015). This lack of follow-up may result from the inherent context-dependent character of ethical leadership, which may limit the appeal and resonance of a more generalist approach. We therefore posit that combining normative and descriptive insights can only turn into practice when sufficient attention is being paid to specific circumstances and values at stake. Furthermore, different sources advocate leadership in sport research to consider leadership as a socially constructed and collective phenomenon, that is co-created by all relevant stakeholders – everyone who impacts or is impacted by the sport organization under study, such as players, coaches, and board members (internal), and sponsors, governments, and the media (external) – involved (Billsberry et al., 2018; Ferkins et al., 2018). In this light, we believe adopting such a stakeholder perspective to study ethical leadership in sport may aid to bridge the gap between the normative and descriptive lines of inquiry, leading to a more comprehensive view on this phenomenon.

## Ethical leadership in sport: A normative perspective

Normative inquiry into ethical leadership has strong historical roots, which go back as far as renowned ancient philosophers such as Plato and Aristoteles (Boaks & Levine, 2017; Takala, 1998). Ever since their written considerations, countless historians and philosophers have been applying a normative perspective on ethical leadership to highlight and discuss leadership’s “potential to greatly benefit or harm the well-being of people” (Ciulla et al., 2018, p. 1). These and other insights on how leadership and ethics are intertwined have been studied in numerous settings, ranging from business, military, and medical contexts to contexts in culture, non-profit organizations, and sport. In sport, knowledge about the connection between leadership and ethics mainly stems from research undertaken in two scientific subdisciplines: sport psychology (studying leadership primarily on or around the sport field, e.g., coaching on the field or in locker rooms) and sport management (examining leadership primarily outside the sport field, e.g., in boardrooms of sport organizations) (Welty Peachey et al., 2015). Supported by (sport) ethics and philosophy insights, both subdisciplines have paid attention to the fact that leading in sport contains an intrinsic ethical dimension DeSensi, 2014; Hancock & Hums, 2015; Welty Peachey et al., 2015).

Several scholars have drawn attention to the relevance and importance of ethical leadership in sport from a normative point of view (DeSensi & Rosenberg, 2010; Grange, 2014). This attention has generated two types of studies: one type that identifies the similarities between ethical leadership in business and in sport, and another type that exhibits the presumed specificity of practicing ethical leadership in sport (Constandt, 2019). Concerning the first type, Bischak and Woiceshyn (2016) have drawn on certain parallels between rock climbing and business, to identify six general ethical leadership virtues, namely rationality, honesty, independence, integrity, justice, and pride. These parallels relate to a shared orientation towards clear goals, a set of diverse challenges, the need for planning, and a risk of failure (Bischak & Woiceshyn, 2016). With regard to the second type, a number of studies has been conducted during the past decade. In a first one, DeSensi (2014) outlined the values of sport (e.g., sportsmanship (sic) and fair play) and stipulated that everyone who is pedagogically involved, holds a responsibility to promote these values in sport, as some kind of laboratory for society. In a second study, Roby (2014) criticized the dominant winning-at-all cost mentality in sport, while highlighting the importance of credible, values-based leadership to create an ethical environment in sport organizations. In a third study, Sagas and Wigley (2014) illustrated the distinction in leadership between doing things right and doing the right things. In a final study, Staurowsky (2014) argued that ethical leadership in sport also consists of honouring the athletes’ rights, an aspect that is currently often neglected.

All these normative studies on ethical leadership in sport (and other studies that do not use the label or lens of “ethical leadership” explicitly) are based on moral reasoning and philosophical inquiry and emphasize that ethical leadership entails the pedagogical promotion of certain values, such as respect, honesty, and fair play. However, it is still unclear whether these (and other) values should be seen as universal and generally applicable (Bauman, 2013). Although an extensive consideration of this debate is beyond the scope of this paper, it can be argued that the interpretation of the values and behaviours that *should* be promoted by leaders to actually be ethical leaders, depends on the unique context at hand and on matching the personal values and preferences of both sides of the leadership relationship (Bauman, 2013; Fehr et al., 2015). In other words, as there is no “one style fits all” approach of ethical leadership, the desired content and implementation of ethical leadership in sport (and beyond) depends on such factors as discipline, time, culture, and prevailing social norms (e.g., equality norms may be interpreted differently across cultures, see Hamm et al., 2008) (Fehr et al., 2015; Heres, 2014).

In summary, the normative perspective on ethical leadership dates back to the long-standing philosophical foundations of leadership inquiry (Ciulla et al., 2018). Its goal is to come to a better understanding of what ethical leadership ought to be and which values it should promote, based on sound and sophisticated moral reasoning (Flanigan, 2018; Price, 2018). Nevertheless, normative ethical leadership research should be on guard that “ignoring empirical realities and simply relying on abstractions and generalities regarding how people ought to behave” might be a dangerous side effect of their scholarly endeavours (Price, 2018, p. 688). Hence, descriptive and empirical social scientific research can help to get a better overview of how ethical leadership is understood in different contexts, as well as of the nature of its antecedents and consequences.

## Ethical leadership in sport: A descriptive perspective

In contrast to the long-lasting roots of the normative and philosophical line of ethical leadership inquiry, the descriptive, empirical, and social scientific line of ethical leadership inquiry is of a more recent date (Brown & Treviño, 2006). Ethical leadership is strongly related with more established leadership conceptualizations, such as transformational, transactional, and servant leadership (Brown & Treviño, 2006). However, since Brown and colleagues’ (2005) seminal work on the topic, many have argued and shown its theoretical and empirical contribution to the field. Brown et al. (2005, p. 120) have offered an often cited working definition of ethical leadership to help guide research: i.e., “the demonstration of normatively appropriate conduct through personal actions and interpersonal relationships, and the promotion of such conduct to followers through two-way communication, reinforcement, and decision-making.”

Since the presentation of this definition, the descriptive and social scientific line of inquiry on ethical leadership has gained momentum in the general management literature, with a multitude of empirical – often quantitative survey based – studies focusing on the antecedents (such as moral identity and emotional stability), mechanisms (such as organizational ethical climate and team cohesion), and consequences (such as followers’ commitment and organizational citizenship behaviour) of ethical leadership (Bedi et al., 2016; Ko et al., 2018; Peng & Kim, 2020). Emphasis has thereby mainly been put on follower perceptions of the leaders’ characteristics and behaviours. Moreover, theoretical support has been found in (a) social learning theory (i.e., people learn most strongly by observing and imitating how their leaders implement and judge certain behaviours and attitudes), (b) social exchange theory (i.e., people feel a certain obligation to reciprocate positive leadership behaviours, such as being respectful and trustworthy), and (c) social identity theory (i.e., people identifying themselves with their leader and organization) (Lawton & Paéz, 2015; Peng & Kim, 2020).

Along with this definition, its underlying theorization, and the development of several survey constructs or scales to measure ethical leadership (see e.g., Brown et al., 2005; Kalshoven et al., 2011; Yukl et al., 2013), prescriptive research has highlighted that three distinct roles can be identified within ethical leadership: (a) being a *moral person* (impersonating such treats, characteristics, and behaviours as being honest, trustworthy, and fair), and acting as a (b) *moral manager* (leading by example, using clear ethics communication and fair reinforcement, while empowering followers), and (c) a *moral entrepreneur* (striving to innovate, by implementing new ethical norms when current norms are insufficient to tackle occurring challenges) (Kaptein, 2019). Offering this definition and identifying these roles, descriptive ethical leadership research has integrated certain normative elements. However, these normative elements remain little elaborated and rather function as a merely structuring element to guide descriptive research without much sound consideration (Flanigan, 2018; Heres, 2014; Price, 2018). Hence, more additional philosophical inquiry and moral reasoning is still needed to enhance our understanding of ethical leadership (Flanigan, 2018; Price, 2018).

Sport sciences have been a considerable late adopter when it comes to studying ethical leadership from an empirical (descriptive) point of view. Part of the explanation for this “delay” in comparison to other fields might reside in sport’s historical preoccupation with (both regulatory and prescriptive) rules (McFee, 2004). In many cases, sport has turned – and is still turning – to regulation and codification to deal with occurring challenges (McFee, 2004). While these formal initiatives are amply needed, they also bear an inherent risk. Leaders of sport organizations regularly tend to hide behind the rules to avoid honouring their responsibilities (e.g., being accountable for their own (lack of) behaviours) (Constandt, 2019; Kihl, 2007; Tomlinson, 2014). However, managing ethics in sport requires an approach that goes beyond mere compliance from a legal and regulative point of view. This approach can be achieved by incorporating mechanisms and initiatives to avoid the occurrence of so-called *loophole ethics* in sport (i.e., the idea that everything that is not explicitly forbidden is implicitly allowed) (Kvalnes & Hemmestad, 2010). After all, fair play in sport entails more than simply following the rules (e.g., by respecting the values and *spirit* of sport) and can only be achieved when formal policies are translated into actual practices by different levels of leadership within sport organizations (Constandt, 2019; De Waegeneer & Willem, 2016).

Today, empirical, descriptive studies on ethical leadership in sport continue to be limited in number and scope (i.e., focusing mainly on college athletics in North America). For instance, Cotrufo (2014) has shown that ethical leadership on behalf of athletic directors can lead to positive organizational behaviour from staff members within college athletic departments. Additionally, Wells and Walker (2016) have outlined the importance of the transparent communication aspect of ethical leadership in a college athletic department during a period of organizational change. Moreover, examining ethical leadership perceptions of 14 US collegiate athletic administrators with the lens of institutional logics, Nite and Bopp (2017) have suggested that these perceptions are shaped by different deeply engrained, yet often incompatible ideals. Nite and Bopp (2017, p. 371) further proposed that (groups of) stakeholders “often have diverse expectations of organizational leadership”. In addition to discussing the role of collegiate athletic administrators, athletic coaches have been shown to impact the moral development, voice behaviour, and performance of their (student) athletes (Hamilton & LaVoi, 2017; White & Rezania, 2019). In particular, drawing on a large survey-based dataset, Yukhymenko-Lescroart et al. (2015) have indicated that coach ethical leadership might positively stimulate athletes’ perceptions of an inclusive team climate, while decreasing their willingness to cheat.

These studies based on North American samples have been supplemented with work on European sport over the past few years. More precisely, Constandt and colleagues (2018) have shown that coach ethical leadership influences the ethical climate in amateur football (soccer) clubs, as well as the affective organizational commitment of football players. Moreover, a trickle-down (or cascading) effect of ethical leadership in amateur football clubs has been exposed, highlighting that the influence of board ethical leadership partly trickles down to the ethical climate of the football club via coach ethical leadership (Constandt & Willem, 2019). Furthermore, broadening the scope to professional football, Constandt, Parent, and Willem (2020) have shown that fans care little about ethical leadership in their club, as they focus mainly on those aspects that impact their own position, such as clear communication and strengthening the fans’ position within the governance of the club. Finally, studying Belgian handball and volleyball settings, De Backer et al. (2018) have indicated a positive influence of a need supportive coaching style on athletes’ perceived justice of their coach.

In most of these studies, ethical leadership in sport has been analysed as an individually perceived phenomenon of which the meaning resides “in the moral eye of the beholder” (Giessner et al., 2015). Particular focus has thereby been put on the perspectives and the perceptions of key internal stakeholders of sport organizations, such as athletic administrators, coaches, and players [for an exception, see the fan study of Constandt and colleagues (2020)]. A couple of authors have acknowledged and advocated the integration of a wider group of internal and external stakeholders in sport leadership research (Billsberry et al., 2018; Ferkins et al., 2018; Kihl et al., 2010). For example, sponsors of sport organizations can demand certain behavioural standards of the leadership of the sport organization they fund, in return for their (continued) support.

## Bridging the gap: Towards a stakeholder perspective on ethical leadership in sport

The normative and the descriptive lines of ethical leadership inquiry have both been the subject of considerable critique. Among other critiques, scholars point out that existing descriptive ethical leadership inquiry is conceptually unclear and incomplete (i.e., concentrating too much on negative reinforcement and too little on such aspects as role clarification, social responsibility, and leader learning), bears limited explanatory power, focuses predominantly on Western cultures [for a few exceptions, see e.g., Dhar, 2016; Garba et al., 2018; Yang, 2014], and has long been measured by means of unidimensional and vague scales (Eisenbeiß, 2012; Eisenbeiß & Giessner, 2012; Flanigan, 2018; Heres et al., 2017; Kalshoven et al., 2011; Shakeel et al., 2020). On the other hand, normative ethical leadership inquiry has been framed as idealistic or even unworldly, lacking actual familiarity with how leadership operates in the real world of business or sport (Harvey, 2001; Price, 2018).

Considering these critiques, future ethical leadership research should move away from the strict division between normative and descriptive studies, but also from the artificial demarcation between classical and contemporary approaches (Shakeel et al., 2020). In essence, both kinds of studies provide “complementary sides of the same coin” (Flanigan, 2018). Therefore, their mutual isolation should be bridged. Normative inquiry helps to stimulate our “understanding of what a leader ought to do” (Flanigan, 2018, p. 707), but different contexts and cultures also imply different prescriptions, expectations, and implementations of ethical leadership (Heres, 2015). As a consequence, offering empirical insights, descriptive inquiry can help to enrich our understanding of the multifaceted nature and context-dependency of ethical leadership as a social and relational phenomenon (Maak & Pless, 2006).

A stakeholder perspective on ethical leadership is promising to bridge this ethical leadership research gap in sport (see Ferkins et al., 2018; Kihl et al., 2010) and beyond (see Eisenbeiß & Giessner, 2012; Heres, 2015). Sport’s complex stakeholder constellation contributes to its uniqueness as a sector, as many different internal (e.g., board members, coaches, players, volunteers) and external (e.g., sponsors, fans, government, media) stakeholders are involved (Walters & Tacon, 2010). However, it is often difficult to define these stakeholders and to determine their individual as well as stakeholder-group related position and salience (Friedman et al., 2004). Drawing on stakeholders’ attributes, such as legitimacy, power, and urgency (see Mitchell et al., 1997), a better picture of the importance of different stakeholder groups can be provided in sport (Friedman et al., 2004). When it comes to ethics and leadership, stakeholders often differ in their perceptions and expectations of ethical leadership, both individually and based on the beliefs, customs, and interests of the stakeholder group(s) they represent (Brown & Mitchell, 2010; Constandt et al., 2020; Eisenbeiß & Giessner, 2012).

Ethical leadership research has already taken a step in the direction of “looking beyond the leader”, by applying a *follower*-centric perspective (i.e., what do followers expect of their leaders in terms of ethical guidance?) instead of a *leader*-centric approach of leadership (i.e., what are the traits and characteristics of ethical leaders?) (Heres, 2015). More precisely, implementing a follower-centric perspective, Heres (2015) has shown that (a) followers differ in their assumptions, expectations, and ideal types of what an ethical leader is/should be, (b) those expectations differ according to the context at hand, and (c) implicit expectations of followers have an impact on the way they interpret the behaviours of their leaders. Therefore, these expectations might influence the effectiveness of implemented leadership behaviours.

Nevertheless, as some people who are confronted with leadership are either non-receptive or not the actual target for its influence – and are thus not actual “followers” but rather “observers” – an *observer*-centric perspective has been proposed (Billsberry et al., 2018). An observer-centric perspective goes further than a follower-centric perspective in that sense that a follower is someone who is having a clear and direct relationship with the leader at hand, while an observer is not necessarily in that position. All followers are observers but not all observers are followers. In this PhD critical review paper, we advocate to go one step further, by adopting a *stakeholder* perspective in which all the above perspectives are integrated and ethical leadership in sport is studied from multiple perspectives simultaneously. Within such stakeholder perspective, attention should be paid to hierarchical and formal views as well as to more horizontal and less formal views on leadership in sport. To clarify, leadership is not the prerequisite of a certain position within a sport organization, as it can also be shown among peers (e.g., team mates).

This stakeholder perspective should be broad, ideally including everyone who impacts or is impacted by the organizational sport setting(s) under study (Freeman, 2010). Moreover, this stakeholder perspective bears both a descriptive (i.e., integrating the perceptions of all relevant stakeholders), as well as a normative (i.e., incorporating the values and prescriptions of all relevant stakeholders) component, while it also looks into how both components continuously impact each other. In sum, this stakeholder perspective provides necessary attention to how the desired and perceived content of ethical leadership is shaped and socially constructed by those involved (Fehr et al., 2015; Ferkins et al., 2018; Heres, 2015).

Such stakeholder perspective not only honours the active nature of ethical leadership as a relational phenomenon that is not merely implemented in a top-down way, but it also re-integrates the perspective of the leaders themselves in ethical leadership research (Maak & Pless, 2006). Acknowledging ethical leadership as a collective and relational phenomenon is required to counteract the idea that followers/stakeholders are just passive and little capacitated followers who are in need of guidance of their leader(s) (Munro & Thanem, 2018). Furthermore, such stakeholder perspective acknowledges both the direct and indirect impact of stakeholders on leadership. After all, operating as “stakewatchers”, more distant stakeholders (e.g., sponsors, fans, parents of minor players) might pressure leaders (e.g., the board of a sport club) indirectly into adapting their relationship with one of their direct followers (e.g., the coaches of the sport club).

This PhD critical review paper argues that there are two main ways to implement a stakeholder perspective on ethical leadership in sport: (a) by conducting and intensifying research in each of the sketched pillars (i.e., leader centric, follower centric, and observer centric research on ethical leadership), and (b) by integrating these three pillars in thorough quantitative and (single and multiple) case study research that aims to compare and contrast findings. Additionally, a more diverse set of stakeholders (including external stakeholders) and sport settings should be considered, while a broader range of methods is required to enhance our understanding of ethical leadership in sport. Finally, these two lines of suggested research should both dedicate sufficient attention to non-empirical, philosophical insights which help to enhance our understanding of ethical leadership based on sound reasoning (Flanigan, 2018; Price, 2018). Existing boundaries between scientific bodies of literature should thereby be overcome, as related fields such as sport psychology, sport management, and sport philosophy insufficiently draw on each other’s complementary leadership insights.

### A more diverse set of stakeholders and sport settings

The importance of a stakeholder perspective on ethical leadership in sport has been highlighted in a study focusing on the extent to which fans care about ethical leadership in professional football (Constandt et al., 2020). However, the viewpoints of many other relevant stakeholders both within and beyond sport – including (national, regional, and local) governments, media, and sponsors – should also be integrated in future work (Hancock & Hums, 2015; Welty Peachey et al., 2015). Paying attention to the influence of distance, be it physically and/or socially, is worthwhile, as it can be an interesting explaining factor when it comes to ethical leadership interpretations (Constandt et al., 2020). Despite their often remote relationship with leaders in sport, stakeholders such as governments and sponsors affect and are affected by sport leadership practices (Hancock & Hums, 2015). For example, these stakeholders can link behavioral or ethical standards to subsidies and sponsor fees, while the media can draw public attention to certain issues. Moreover, it is worthwhile to investigate how specific scandals impact both perceptions and prescriptions of ethical leadership in sport.

Furthermore, forthcoming scholarship on ethical leadership in sport should broaden its scope to more diverse settings. Currently, both normative and descriptive insights in ethical leadership are quite limited to intercollegiate athletics in North America and football (soccer) in Europe. To expedite comparisons, other sports and regions should be explored to examine whether context-dependent differences are really present. In other words, how much common ground (i.e., common understanding and meaning) is present about ethical leadership in sport? Additionally, we encourage future work to pay attention to the extent of stakeholder group homogeneity vs. heterogeneity (i.e., the degree to which people belonging to the same stakeholder group think alike concerning ethical leadership) and to the role of trust and values. When assessing ethical leadership, it is particularly interesting to examine whether stakeholders are actually rating their leaders’ ethical leadership or rather the extent in which they actually like, trust, and/or share the values of their leaders (Heres, 2015).

While broadening the scope of ethical leadership in sport research to more diverse settings, attention could be paid to more practice-oriented research questions and to the practical implications of the study’s findings. Studies guided by such research questions as “How could ethical leadership be increased among coaches?” and “How can coaches be ethical yet demanding in elite sport contexts, and what are the underlying mechanisms?” could help to stress the practical relevance of ethical leadership in sport scholarship in a clearer way. Moreover, such research would support the evidence-based development of specific valorization tools (e.g., dilemma training in sport clubs), aimed at stimulating ethical leadership in sport practices.

### A broader range of methods

In terms of methods, future work is encouraged to strengthen the current dominant yet limited quantitative ethical leadership measurement scales. After all, these generic scales hold inherent biases that measure not only the behaviours that people observe in leaders, but also the respondents’ own prior expectations, ideals, and assumptions of ethical leadership, as well as their general trust in their leader (Heres, 2015; van den Akker et al., 2009). In light of these limitations, future quantitative ethical leadership in sport studies are encouraged to develop tailor-made, robust, and validated measurement scales that incorporate the opinions of as many stakeholders as possible. This would help to avoid conceptual vagueness and confusion when it comes to ethical leadership in sport. It would also facilitate comparing different sports and rendering practical suggestions.

In addition to developing and implementing these scales on ethical leadership (in sport), the use of qualitative methods that enable more fine-grained work on ethical leadership is amply needed to examine (a) what is actually understood by ethical leadership in different settings and by different stakeholders, (b) how these understandings influence stakeholders’ actions, and (c) what the desired and supported comprehension of ethical leadership is in a given context (Ko et al., 2018). Evidently, sufficient philosophical consideration has to be integrated in all three applications to analyse how sense can be made of such an abstract and complex phenomenon as ethical leadership.

A broader range of methods is thus required to respect the complex nature of ethical leadership in sport. The multiple interactions between different stakeholders, both on and of the sport field, should thereby be taken into consideration. For example, (a) participatory observation in sport organizations, (b) social network analysis (SNA), (c) and novel methods such as smartphone apps could be specific applications of future ethical leadership research. Participatory observation and smartphone apps facilitate the collection and analysis of real time and recurrent responses when it comes to certain sport contexts (e.g., fans’ or sponsors’ viewpoints on ethical leadership during a sport competition). Such apps might thus support the measurement of the evolution of ethical leadership over time, analysing the (un)stability of ethical leadership as a construct. Furthermore, SNA could help to shed light on the role of power distributions and underlying ties between stakeholders (Claringbould et al., 2018; Eisenbeiß & Giessner, 2012; García et al., 2016). SNA thus aids the assessment of leadership as the complex, relational, and collective phenomenon it is. As sport is usually taking place in a competitive environment, these methods might also help to scrutinize whether ethical leadership perceptions (i.e., a descriptive view) and prescriptions (i.e., a normative view) are related with the (lack of) success on the sport field (Constandt et al., 2020).

## Conclusion

As a unique leisure time activity and branch of the entertainment industry, sport is often offering a stage for people to showcase both the best and worst kinds of human behaviours. While sport is believed to stimulate health and social cohesion and to bring joy, numerous indications of different ethical issues are surrounding sport’s manifestations. In this light, ethical leadership is considered to be part of the desired approach within sport organizations to mitigate this tension. Nonetheless, the scope of existing scholarship on ethical leadership in sport is not yet that broad, leaving debates on two important and context-dependent questions largely unsettled: (a) how should ethical leadership look like (i.e., a normative question focusing on prescriptions)?, and (b) how is ethical leadership understood in practice (i.e., a descriptive question, focusing on perceptions)? Despite their interplay, both research questions are currently mainly assessed in certain isolation of each other. This PhD critical review paper therefore advocates forthcoming studies to integrate both questions by means of a broad stakeholder perspective. Such stakeholder perspective would help to learn how all people involved in sport (both internal and external to the sport organization(s) under scrutiny) help to make sense of ethical leadership in sport. Moreover, such perspective would also help to guide sport organizations’ actions to successfully overcome the ethical challenges they are facing.

## Footnotes

For the purpose of this paper, ethics and morality are considered synonyms.
